# Association between atrial fibrillation and *Helicobacter pylori*

**DOI:** 10.1007/s00392-019-01418-w

**Published:** 2019-02-08

**Authors:** Cecilia Tetta, Amalia Ioanna Moula, Francesco Matteucci, Orlando Parise, Bart Maesen, Daniel Johnson, Mark La Meir, Sandro Gelsomino

**Affiliations:** Department of Cardiothoracic Surgery, Cardiovascular Research Institute Maastricht -CARIM, Maastricht University Medical Centre, Universiteitssingel 50, 6229 ER Maastricht, The Netherlands

**Keywords:** Atrial fibrillation, Helicobacter pylori, Metanalysis

## Abstract

The connection between atrial fibrillation (AF) and *H. pylori* (HP) infection is still matter of debate. We performed a systematic review and metanalysis of studies reporting the association between AF and HF. A systematic review of all available reports in literature of the incidence of HP infection in AF and comparing this incidence with subjects without AF were analysed. Risk ratio and 95% confidence interval (CI) and risk difference with standard error (SE) were the main statistics indexes. Six retrospective studies including a total of 2921 were included at the end of the selection process. Nine hundred-fifty-six patients (32.7%) were in AF, whereas 1965 (67.3%) were in normal sinus rhythm (NSR). Overall, 335 of 956 patients with AF were HP positive (35%), whereas 621 were HP negative (65%). In addition, 643 of 1965 NSR patients (32.7%) were HP positive while 1,322 were negative (67.3%; Chi-square 2.15, *p* = 0.21). The Cumulative Risk Ratio for AF patients for developing an HP infection was 1.19 (95% CI 1.08–1.41). In addition, a small difference risk towards AF was found (0.11 [SE = 0.04]). Moreover, neither RR nor risk difference were influenced by the geographic area at meta-regression analysis. Finally, there was a weak correlation between AF and HP (coefficient = 0.04 [95% CI −0.01–0.08]). We failed to find any significant correlation between *H. pylori* infection and AF and, based on our data, it seems unlikely than HP can be considered a risk factor for AF. Further larger research is warranted.

## Introduction

Despite better knowledge of the pathophysiology of atrial fibrillation(AF) [1–17] and significant advances in ablation techniques [18–26], its incidence is rising worldwide [27], resulting in a dramatically increased social and economic burden on the healthcare system [28–31].

The pathophysiology of AF is complex [32] and it has been found to be associated with inflammation as well as other non-cardiac pathologies [33–35]. Among these, gastrointestinal (GI) disorders share with AF some risk factors such as stress, smoking and inflammation as well as some common symptoms such as chest pain and faintness [36] and they have often been described in association with AF [37].

In particular, *Helicobacter pylori* (HP) infection has been strongly linked to AF by previous studies leading to the hypothesis that HP could be the cause of AF through systemic inflammatory response [37–39]. Nonetheless, this connection has been questioned by other authors and it is still matter of debate [40, 41]. Furthermore, available metanalyses include other supra-ventricular arrhythmias other than AF [42] or include other infections associated to AF [39].

Therefore, to test the hypothesis whether there is a causal relation between AF and the infection of HP, we performed a systematic review and metanalysis of the studies published reporting this association.

## Materials and methods

### Search strategy

Literature search was conducted in accordance with the Preferred Reporting Items for Systematic Review and Metanalyses (PRISMA) [43]. An unrestricted literature search was performed using PubMed, EMBASE, Web of Science and Google Scholar Databases, as well as well as congress proceedings from major cardio cardiothoracic and cardiology societies meetings.

Search terms were: “Atrial Fibrillation” OR “Atrial Fibrillation AND Gastritis” OR "Atrial Fibrillation AND Helicobacter pylori”, OR “Atrial Fibrillation AND stomach” OR “Atrial Fibrillation AND Digestive Disease” OR “Atrial Fibrillation AND Digestive Disorders” OR “Arrhythmias AND Gastritis” OR "Arrhythmias AND Helicobacter pylori”, OR "Arrhythmias AND stomach” OR "Arrhythmias AND Digestive Disease” OR "Arrhythmias AND Digestive Disorders”.

The search strategy was decided by two authors (C.T. and A.I.M.) and approved by another reviewer (B.M.). The literature was limited to articles published in English. References of original articles were reviewed manually and cross-checked for other relevant reports.

## Selection criteria and quality assessment

Studies were included if they met all of the following criteria: (1) human studies; (2) full articles about AF and HP having a non-AF control population; (3) adequate information regarding the positivity to HP infection. Exclusion criteria were: (1) animal studies; (2) case report; reviews; (5) lack of information for meta-analysis.

Two authors (A.I.M. and C.T.) selected the study for inclusion, extracted studies, as well as patient information and outcomes. Two reviewers (S.G. and M.L.M.) independently assessed eligibility of the studies and risk of bias. Risk of bias at the individual study level was assessed using ROBINS-I tool (Risk of Bias in No-randomized Studies-of Interventions) [44].

## Methodological quality assessment

The quality of included studies was assessed using a rating scale based on Downs and Black’s Checklist for Measuring Quality [45]. This rating scale for non-randomized designs was recently adapted for use in meta-analytic research on interpretation biases toward illness-related information [46]. The ratings scale consisted of 18 items assessing the quality in terms of reporting, external validity, internal validity, confounders, and power of the study. Each criterion is rated on a two-point scale (0/no, 1/yes), with exception of item 11 (“confounders described and controlled for”) that has a score ranging from 0 to 2, with higher scores indicating superior quality. Two independent researchers (F.M. and O.P) conducted the ratings. Any divergences were resolved by a third reviewer (B.M) and quantified using the Cohen’s kappa [47, 48].

### Endpoints

The primary endpoint was HP infection defined as diagnosed infection either by biopsy-based tests including histological evaluation, culture, polymerase chain reaction (PCR), and rapid urease test (RUT) or non-invasive procedure such as breath test (UBT), serology, and stool antigen test (SAT) [49].

### Statistical analysis

Meta-analysis was conducted using Comprehensive Metanalysis v.2.2 (Biostat, Englewood, New Jersey) and Stats Direct v.3.0 (Stats Direct Ltd Cambridge, UK). Risk ratio and 95% confidence interval (CI) and risk difference with standard error (SE) were the main statistics indexes for binary outcomes. The statistical inconsistency test I^2^ was used to assess heterogeneity [50]. Nonetheless, because the high degree of heterogeneity anticipated among the available studies (only non-randomized trials) and inverse variance (DerSimonian Laird) an inverse-variance-random effect model was employed as a more conservative approach accounting for between- and within-study variability. Publication bias was evaluated graphically using a funnel plot and determined mathematically using Egger regression and the Begg–Mazumdar rank correlation test.

A correlation analysis was carried out using the Schmidt–Hunter method that provides the least biased estimate [51]. In addition, by means of meta-regression, the impact of the geographic area across single studies and its relationship to the occurrence of the primary endpoint was investigated. All p values < 0.005 were considered statistically significant.

## Results

### Characteristics of the studies

**Fig. 1:**
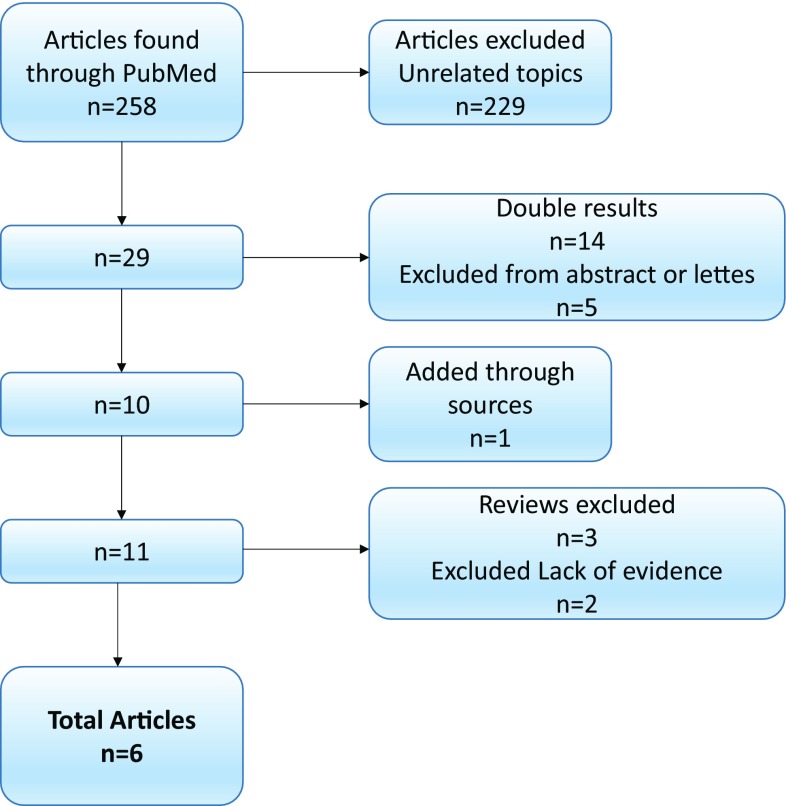
PRISMA diagram of the study selection process

The PRISM flow diagram describing the study selection process along with reasons for exclusion is shown in Fig. 1. The number of studies found was 258. Of these articles 229 were excluded as not related to the topic. Twenty-nine articles underwent further screening after exclusion of 14 double results, 10 were found to be on topic and suitable for further evaluation. One article was added from the references of the sources. Subsequently, 11 articles were further assessed. Of those four review articles and two articles that did not have appropriate end points and/or missing statistical data were excluded. After removal of reports not pertinent to the design of the current metanalysis, six retrospective observational studies that met explicit inclusion criteria remained, including a total of 2,921 patients [40, 41, 52–56]. Nine hundred-fifty-six patients (32.7%) were in AF, whereas 1965 (67.3%) were in normal sinus rhythm (NSR). Detailed characteristics of studies and patients are described in Table 1.

**Table 1:** Patient characteristics

	Wang et al. 2010			Ki et al. 2009		Lunetta et al. 2009		Bunch et al. 2008 (*n* = 83)		Platonov et al. 2007		Badran. et al. 2007	
	AF (*n* = 285)		Controls (*n* = 300)	AF (*n* = 60)	Controls (*n* = 36)	AF (*n* = 39)	Controls (*n* = 141)	AF (*n* = 83)	Controls (*n* = 660)	AF (*n* = 72)	Controls (*n* = 72)	AF (*n* = 82)	Controls (*n* = 80)
	NLSP	LSP											
Age	62.1 ± 11.78	65.1 ± 9.80		61.28 ± 1.35	50.64 ± 2.18	“About 64”	“About 64”	70.9 ± 9.5	63.9 ± 10.7	69.6 ± 8.3	69.8 ± 7.5	62 ± 4	66 ± 3
Sex	82 (65.1)	93 (58.5)	198(66)	23 (38)	21 (58)	–	–	20 (24)	154 (23)	23 (31.9)	20 (27.8)	37 (45.1)	32 (40.0)
Smoking	52.4	50.9	53	–	–	–	–	25	29	–	–	–	–
Hypertension	78.6	76.1	73.7	–	–	–	–	58	53	–	–	–	–
Dyslipidemia	43.7	39.0	37.3	–	–	–	–	48	51	–	–	–	–
Diabetes mellitus	25.4	34.0	30.0	–	–	–	–	21	18	–	–	–	–
CVA	29.4	34.0	27.3	–	–	–	–	–	–	–	–	–	–
Family history of AF	5.6	3.1	3.7	–	–	–	–	–	–	–	–	–	–
LVEF	61.9 ± 10.80	60.8 ± 10.55	61.4 ± 10.13	–	–	–	–	–	–	–	–	46.7 ± 10.5	–
LAD	34.8 ± 4.79	36.7 ± 5.86	32.9 ± 4.45	–	–	–	–	–	–	–	–	39.1 ± 0.9	–
BMI	25.2 ± 3.41	25.5 ± 4.16	25.5 ± 3.88	–	23.92 ± 0.55	–	–	28.5 ± 5.4	29.6 ± 15.8	–	–	–	–
Median hs-CRP	[0.8]	[1.26]	[8.0]	–	–	–	–	-	-	–	–	–	–
CRP	–	–	–	0.12 ± 0.11	0.05 ± 0.11	–	–	2.2 ± 2.7	2.3 ± 2.4	–	–	2.81 ± 2.87	0.97 ± 1.02
Median HCY	11.9	12.60	9.90	–	–	–	–	–	–	–	–	–	–
TNF-α	–	–	–	1.39 ± 0.24	0.13 ± 0.21	–	–	–	–	–	–	–	–
IL-6	–	–	–	3.75 ± 0.67	2.62 ± 0.49	–	–	–	–	–	–	–	–
TGF-β1	–	–	–	104.18 ± 26.63	204.00 ± 42.79	–	–	–	–	–	–	–	–
Beta blockers	–	–	–	–	–	–	–	57	49	18	0	–	–
Statin	–	–	–	–	–	–	–	13	18	–	–	–	–
Digoxin	–	–	–	–	–	–	–	–	–	35	0	–	–
Calcium channel blockers	–	–	–	–	–	–	–	–	–	15	0	–	–
Diuretics	–	–	–	–	–	–	–	–	–	29	0	–	–
ACE-inhibitors	–	–	–	–		–	–	10	20	7	0	–	–

Numeric data are reported as mean ± standard deviation or [median]. Categorical data are reported as number and (%)

*NLSP* no long-standing persistent AF (defined short-standing by the authors), *LSP* long-standing persistent AF, *SBP* systolic blood pressure, *CVA* cerebrovascular accident, *Bpm* beats per minute, *WBC* white blood cells, *NEU* neutrophil, *LVEF* left ventricular ejection fraction (%), *LAD* left atrium diameter (mm), *BMI* body mass index (kg/m^2^), *hs-CRP* high sensitive C-reactive protein (mg/dL), *HCY* homocysteine (µmol/L), *Hp Helicobacter pylori, TNF-α* tumor necrosis factor alpha, *IL-6* Interleukin 6 (pg/mL), *TGF-β1* transforming growth factor beta 1 (pg/mL), *ACE* angiotensin-converting-enzyme, *CRP* C-reactive protein (mg/dL)

#### Methodological quality

The average overall quality rating was 0.81 ± 0.26 with ratings ranging from 0.58 to 1. Table 2 presents the average scores on the items of the checklist. The table reveals lower scores for the item assessing whether the studies tested participants’ engagement with the task(s) and items related to the quality of reporting (confounders, exact probability values, withdrawals/drop-outs, and power analysis). Acceptable inter-rater agreement was found (*κ* = 0.74).

**Table 2:** Quality assessment

	Item	*M*	SD
1	Study hypothesis/aim/objective described?	1.00	0.00
2	Main outcomes described in the introduction or methods?	0.97	0.16
3	Participant characteristics described?	0.92	0.27
4	Contacted participants representative?	0.87	0.33
5	Prepared participants representative?	0.86	0.35
6	Participants recruited from the same population?	0.70	0.46
7	Participants recruited over the same time?	0.97	0.16
8	Measures and experimental tasks described?	1.00	0.00
9	Main outcome measures valid and reliable?	1.00	0.00
10	Task engagement assessed?	0.33	0.50
11	Confounders described and controlled for?	0.58	0.65
12	Statistical tests appropriate?	1.00	0.00
13	Main findings described?	0.97	0.16
14	Estimates of the random variability in data main outcomes?	0.86	0.35
15	Probability values reported?	0.58	0.50
16	Withdrawals and drop-outs reported?	0.61	0.49
17	Data dredging made clear?	0.92	0.27
18	Sufficient power analysis provided?	0.80	0.27

All items have a maximum score of 1.00 except item 11 has maximum score of 2.00

#### Main endpoint

**Fig. 2:**
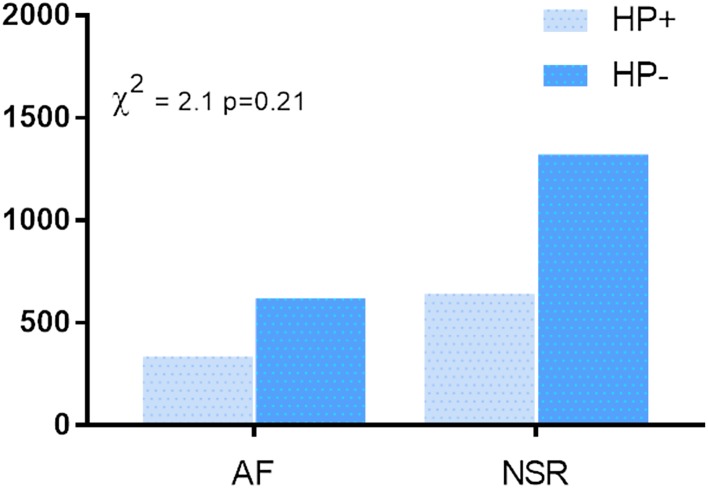
*H. pylori* infection in patients with and without AF

All the seven selected studies contributed to the analysis. Table 3 shows HP diagnostic criteria. In the overall analysis (Fig. 2), 335 of 956 patients with AF were HP positive (35%), whereas 621 were HP negative (65%). In addition, 643 of 1,965 NSR patients (32.7%) were HP positive while 1322 were negative (67.3%; *χ*^2^ 2.15, *p* = 0.21).

**Table 3:** *Helicobacter pylori* infection data

	Wang et al. 2010			Ki et al. 2009		Lunetta et al. 2009		Bunch et al. 2008 (*n* = 83)		Platonov et al. 2007		Badran. et al. 2007	
	AF (*n* = 285)		Controls (*n* = 300)	AF (*n* = 60)	Controls (*n* = 36)	AF (*n* = 39)	Controls (*n* = 141)	AF (*n* = 83)	Controls (*n* = 660)	AF (*n* = 72)	Controls (*n* = 72)	AF (*n* = 82)	Controls (*n* = 80)
	NLSP	LSP											
Hp-IgG antibody positive	60 (47.6)	79 (49.7)	131 (43.7)	36 (60.0)	12 (33.3)	25 (64.1)	95 (67.4)	54 (65.1)	362 (54.8)	41 (56.9)	40 (55.6)	–	21 (26.3)
Hp-IgG antibody	–	–	–	19.86 ± 2.63	13.69 ± 4.12	–	–	–	–	–	–	–	–
Hp-δ value median	[0.700]	[1.90]	[0.625]	–	–	–	–	–	–	–	–	–	–
Hp-δ value ≥ 4‰	24.6	81.8	28.7	–	–	–	–	–	–	–	–	–	–
CagA Hp-IgG antibody positive	–	–	–	–	–	–	–	–	–	–	–	52 (63.4)	21 (26.3)
Anti-Vac-A IgG	–	–	–	1.50 ± 3.49	1.03 ± 1.37	–	–	–	–	–	–	–	–

Numeric data are reported as mean ± standard deviation or (median). Hp-IgG antibody positivity is reported as number and (%). Categorical data are reported as %

*Hp IgG antibody Helicobacter pylori* immunoglobulin G antibody (mg/L), *CagA Hp-IgG antibody Helicobacter pylori* virulence factor CagA (cytotoxin-associated gene A) immunoglobulin G antibody, *Anti-Vac-A IgG (mg*/*L)* anti-*Helicobacter pylori* vacuolating cytotoxin A

**Fig. 3:**
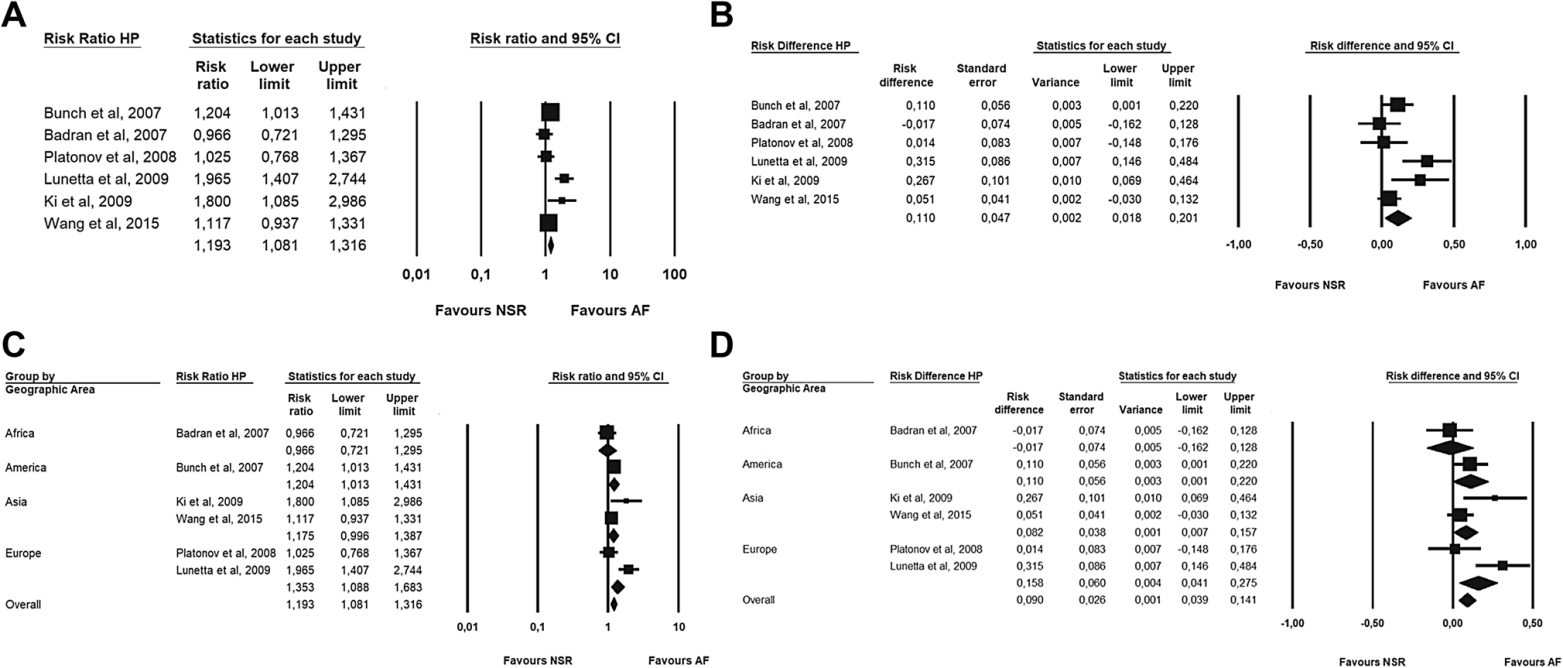
Forest plot. **a** Risk ratio of HP in patients with AF. **b** Risk difference of HP incidence between patients with or without AF. **c** Risk ratio of HP in patients with AF by geographic area. **d** Risk difference of HP incidence between patients with or without AF by geographic area

The cumulative risk ratio (Fig. 3a) for AF patients for developing an HP infection was 1.19 (95% CI 1.08–1.41). In addition, a small difference risk (Fig. 3b) towards AF was found (RD = 0.11 [SE = 0.04]). Moreover, neither RR nor risk difference was influenced by the geographic area (Fig. 3c, d) at meta-regression analysis.

**Fig. 4:**
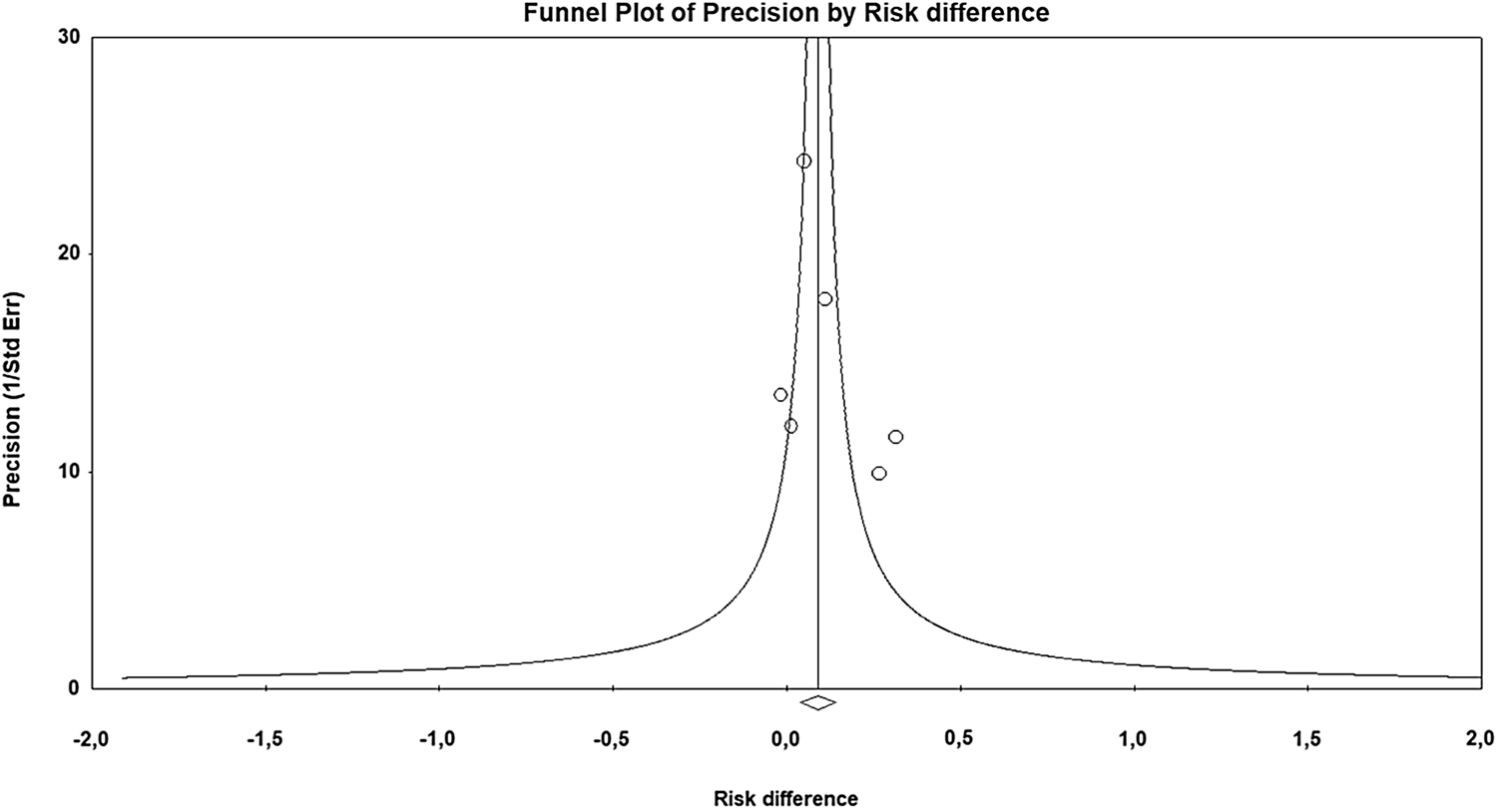
Funnel plot

There was significant heterogeneity in the selected studies (*I*^2^ = 63.6%, *p* = 0.011), thus the random-effect model was employed. No publication bias was observed in the funnel plot **(**Fig. 4**)** and as confirmed in Egger’s test for the asymmetry (intercept − 0.91, 95% CI − 3.8–5.7, *p* = 0.62) and Begg and Mazumdar test (*τ* = − 0.04, *p* = 0.88) that were not significant.

**Fig. 5:**
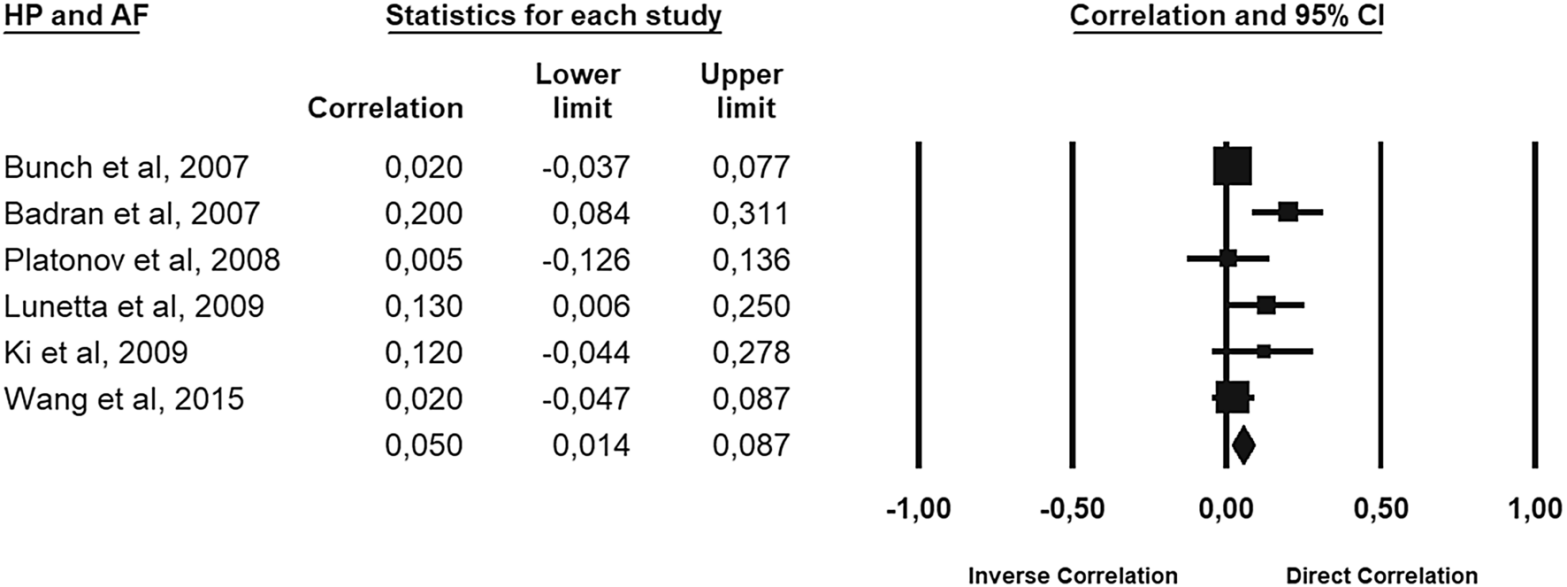
Correlation between AF and HP

Finally, there was a weak correlation (Fig. 5) between AF and HP (coefficient = 0.04 [95% CI − 0.01–0.08]). There was significant heterogeneity in the selected studies (*I*^2^ = 48%, *p* = 0.02) thus the random-effect model was employed (Egger’s test, intercept 1.5, 95% CI − 1.8–4.9, *p* = 0.30; Begg and Mazumdar test, *τ* = − 0.23, *p* = 0.45).

## Discussion

*Helicobacter pylori* is a Gram-negative bacterium affecting nearly half of the world’s population [49], but only a small percentage of infected patients develop more severe pathologies, such as ulcers (10–15%) and stomach adenocarcinomas (less than 1%) [57, 58].

The association between atrial fibrillation (AF) and *Helicobacter pylori* (HP) infection was first reported by Montenero et al. in 2005 who found higher levels of IgG antibodies in patients with AF compared to healthy volunteers [37].

This association was believed to be on an inflammatory basis and it has been postulated that the infection might be the substrate of the systemic inflammation manifesting in AF [38]. Indeed, there is strong evidence to support the influence of inflammation in the pathogenesis of atrial fibrillation [59] which is associated with increased levels of markers which reflect an underlying inflammatory process [60]. Actually, the levels of high-sensitivity C-reactive protein (hs-CRP) have been shown to be higher among patients with AF compared with controls in sinus rhythm [60] and the stronger association of HP and AF in patients with persistent AF [37] along with the demonstration that persistent AF patients have higher hs-CRP levels than paroxysmal AF patients [61], would further support the involvement of HP in the chronic atrial inflammation resulting in AF. However, in contrast with these authors, Marcus et al. [62] failed to find any association between atrial fibrillation and all inflammatory indices (C-protein, TNF-alpha, CD40 ligand, monocyte chemoattractant protein 1, fibrinogen) excluding IL-6. A recent study [63] has explained that the development of atrial cardiomyopathy, the results of complex of structural, architectural, contractile or electrophysiological changes affecting the atria, creates a substrate which can maintain AF. Nonetheless, not one specific risk factor but multiple concomitant modifiable cardiovascular risk factors determine the manifestation and progression of AF. Recent papers identify the alteration of the gut microbiota (dysbiosis) in most cardiovascular AF risk factors being responsible of AF progression through derived metabolites that affect atrial remodeling [64, 65]. Nonetheless, whether and how dysbiosis might contribute to atrial remodeling and AF progression remains unknown.

Regarding the causal pathophysiologic mechanism, it has been postulated that, due to the similarity between H+/K+-ATPase of gastric and cardiac cells, autoantibodies to H+/K+-ATPase would hamper the ATP hydrolysis and that this unbalance would trigger AF by determining depolarization delay and inducing premature atrial contractions [37].

The association of HP and AF was reported also by Whang et al. [56] in a Chinese population of 285 patients and by Franceschi et al. [55] who found an epidemiological link between HP and supraventricular dysrhythmias, including AF, and ventricular arrhythmias in 54 arrhythmic patients compared to 50 healthy controls. Finally, Bunch et al. [52] found that patients with AF were more likely to be seropositive for HP than the non-AF-control group. Furthermore, these authors report that younger patients (< 50 years) showing a higher risk (8%) of AF of those who were HP seropositive. Interestingly, although the oldest group had the highest overall incidence of AF, there was, among them, only a small increase in risk if they were seropositive for HP.

However, the association between HP and AF has been strongly argued by other studies and available reviews and metanalyses have not helped to solve the matter because of their limitations [39, 42].

For instance, Platonov et al. [41] who have reported no significant association between atrial fibrillation and H. pylori infection in 72 patients with permanent AF compared 1:1 to controls, despite significantly higher levels of C-reactive proteins in these subjects. These authors stopped the patient recruitment for the study due to a constant reduction in differences between groups. In addition, Lunetta et al. [40] showed only a small difference in developing AF between HP-positive and HP-negative subjects (21% vs. 18%) thus excluding that inflammation induced from HP might be responsible of new-onset AF.

Our analysis confirms these findings: indeed, the pooled HR was very close to one with a 11% risk difference that it can be easily influenced by other factors not analyzed by papers (sex, familiarity, obesity, race, etc.). Furthermore, we found a weak correlation between the infection of HP and the development of AF (coefficient = 0.05).

Therefore, from our data, it seems that there is no strong pathogenic link between the bacterial infection and the atrial arrhythmias. Hence, it seems more likely that remodeling and atrial damage lead to an increase in C-reactive protein rather than the opposite. This is also supported by the demonstration that such an increase occurs already at first AF onset and in paroxysmal AF [66], thus apparently excluding any etiopathogenetic role of the HP.

However, if further studies confirm that HP is not responsible either for the initiation or the maintenance of AF, in accordance to our findings, the proposed eradication of HP infection as possible treatment for AF patients, proposed by some authors [38], it would not be even an option for these patients not only for its low cost-effectiveness, but also for the risk to further spread antibiotic resistance.

However, when HP arrives in the human stomach, it may penetrate the mucin layer and adhere to the gastric epithelial cells or it may pass through the stomach without colonizing the mucosa. In the stomach, after initial colonization, many chemical, biochemical, and immunologic reactions take place that are of importance in the progress of the infection and the development of disease [67].

In the major part of cases, infections are chronic, whereas acute *H. pylori* disease, lasting for a few weeks, and characterized by abdominal pain and infiltration of polymorph nuclear leucocytes in the gastric mucosa, is rarely described [67]. All papers included in this review reported HP-IgG-positivity, therefore we can assume that in all these patients B lymphocytes were activated by antigen-presenting cells and that a humoral immune response to *H. pylori* was initiated as response to HP infection.

Finally, an interesting finding of our metanalysis is that, in contrast with previous papers [42], the risk ratio of HP was not influenced by the geographic area. This difference might be due to the inclusion by these authors of one study including patients with idiopathic dysrhythmias, leading them to different conclusions. Said that our findings need to be read with caution since the small number of studies coming from Africa and America, these are not surprising. Indeed, the *H. pylori* infection rate is higher in Asia and Africa than that in western countries, in relation to different standards of hygiene and socioeconomics [5], whereas AF shows higher prevalence, disability-adjusted-life years (DALYs) and mortality in high-income countries than low-middle income and developing countries. This difference is significant, but it must be interpreted with caution since it might be related to under-reporting, limited access to health care services and geographically disparity in published data [27, 68, 69].

Finally, the higher number of studies coming from western countries might justify the high AF prevalence reported (> 30%) and definitely higher than expected in the general population. However, inter-studies heterogeneity also regarding AF prevalence variability was addressed by the random-effect model used for metanalysis.

### Study limitations

This metanalysis has some limitations that have to be pointed out.

Firstly, the number of studies is small, and all papers were case–control studies with high-degree of heterogeneity. Secondly, the number of studies in different geographic areas is too small to allow us to draw any final conclusion. Third, different diagnostic methods were employed for the diagnosis of HP infection and it is not reported if the infection is active. Fourth, only a few papers reported the analysis by AF type and this did not allow a subgroup analysis. Fifth, monitoring and detection of AF was not specified in the studies.

## Conclusions

In conclusion, we did not find any significant correlation between *H. pylori* infection and AF and, based on our data, it seems unlikely than HP can be considered a risk factor for AF. Further larger research is warranted to establish a potential role of HP in the pathophysiologic development of AF.
